# Current Status of Implantable Cardioverter‐Defibrillators (ICDs)/Cardiac Resynchronization Therapy With Defibrillators (CRT‐Ds) for Primary Prevention of Sudden Cardiac Death

**DOI:** 10.1002/joa3.70349

**Published:** 2026-04-23

**Authors:** Hisashi Yokoshiki, Masaya Watanabe, Takeshi Mitsuhashi, Akihiko Shimizu

**Affiliations:** ^1^ Department of Cardiovascular Medicine Sapporo City General Hospital Sapporo Japan; ^2^ Department of Cardiovascular Medicine Caress Memorial Hospital Sapporo Japan; ^3^ Department of Cardiovascular Medicine Hoshi General Hospital Kōriyama Japan; ^4^ UBE Kohsan Central Hospital Ube Japan

**Keywords:** cardiac resynchronization therapy with a defibrillator (CRT‐D), electrophysiological study (EPS), heart failure with reduced ejection fraction (HFrEF), implantable cardioverter‐defibrillator (ICD), out‐of‐hospital cardiac arrest, primary prevention of sudden cardiac death

## Abstract

Among recipients of implantable cardioverter‐defibrillators (ICDs) or cardiac resynchronization therapy with defibrillators (CRT‐Ds), non‐ischemic etiology is predominant, accounting for more than 60% of cases in Japan, which is higher than that in the United States and other European countries. Despite recent concerns about primary prevention ICD/CRT‐D implantation for non‐ischemic patients with systolic heart failure in advanced guideline‐directed medical therapy (GDMT), ICD/CRT‐D therapy is likely effective in reducing the slope of the relationship between all‐cause death and sudden cardiac death. Two cohort studies in Japan have shown that the cumulative incidence of appropriate ICD/CRT‐D therapy is higher among non‐ischemic patients than ischemic patients receiving primary prevention ICD/CRT‐D implantation. Additionally, recent published cohort studies in the United States, Europe, and Japan reported a significant decrease in the risk of all‐cause death in primary prevention ICD/CRT‐D recipients compared to those without ICD/CRT‐D therapy. Notably, ICD/CRT‐D utilization in Japan is the lowest among the Group of Seven (G7) countries. Governmental acknowledgment of the value of primary prevention ICD/CRT‐D therapy, as well as educational activities for non‐cardiac electrophysiologists, are required to prevent a potential labor shortage and an excess of healthcare costs following out‐of‐hospital cardiac arrest (OHCA).

## Graphical abstract

Among recipients of ICDs/CRT‐Ds, the proportion of non‐ischemic cardiomyopathy is more than 60% in Japan. Despite recent concerns about primary prevention ICD/CRT‐D implantation for non‐ischemic patients with HFrEF in guideline‐directed medical therapy, ICD/CRT‐D therapy is likely effective in reducing the slope of the relationship between all‐cause death and sudden cardiac death.

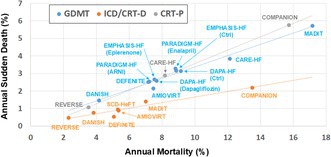

## 1 Introduction

Implantable cardioverter‐defibrillators (ICDs) prevent sudden cardiac death in patients with systolic heart failure and a left ventricular ejection fraction (LVEF) of 35% or less, as well as a New York Heart Association (NYHA) classification of II or III, who have no history of sustained ventricular tachycardia (VT) or fibrillation (VF) [1, 2]. Since 1996, reimbursement of ICD implantation for the primary prevention of sudden cardiac death in Japan has been restricted to patients with inducible VT/VF by electrophysiological study (EPS), as demonstrated by the Multicenter Automatic Defibrillator Implantation Trial (MADIT) [3]. MADIT showed that prophylactic therapy with an ICD improved survival compared to conventional medical therapy in patients with a prior myocardial infarction, LVEF ≤ 35%, and a documented episode of asymptomatic non‐sustained ventricular tachycardia (NSVT), as well as inducible VT/VF by EPS. Subsequently, ICDs reduced all‐cause mortality in ischemic patients with LVEF ≤ 30% (MADIT II) [4] and in heart failure patients with LVEF ≤ 35% and NYHA classification II or III (SCD‐HeFT) [1]. However, the Danish Study to Assess the Efficacy of ICDs in Patients with Non‐ischemic Systolic Heart Failure on Mortality (DANISH) showed that primary prevention ICD implantation in non‐ischemic patients with symptomatic systolic heart failure was not associated with a significantly lower rate of all‐cause death than usual clinical care [5].

This review article aims to briefly summarize the current status and effectiveness of primary prevention ICD/cardiac resynchronization therapy with a defibrillator (CRT‐D) implantation, as well as its impact on healthcare expenditures in Japan.

## 2 Role of Electrophysiological Study (EPS) for Identification of Patients at Risk of VT/VF

The Multicenter Unsustained Tachycardia Trial (MUSTT) demonstrated that, among ischemic patients with a LVEF ≦ 40% and NSVT, those with inducible sustained VT by EPS were associated with significantly increased risk of cardiac arrest or arrhythmic death, as compared with those with non‐inducibility. On the other hand, the rate of these arrhythmic events was still 12% at 2 years, and was not negligible [6]. The sub‐analysis of the MUSTT confirmed that both low LVEF and inducibility by EPS were significant risk factors for total mortality and arrhythmic death. However, the rate of arrhythmic death was not significantly different between non‐inducible and inducible patients with a LVEF < 30% [7]. Thus, we would expect greater predictive utility of EPS in patients with a LVEF more than 30%.

Electrophysiologic inducibility in the MADIT II patients was not associated with an increased likelihood of spontaneous VT/VF. The 2‐year point estimate of the incidence of at least one therapy for either VT or VF was 29.4% and 25.5% respectively. With analysis of ICD treatment for VT or VF separately, inducible patients were significantly more likely to experience a first appropriate ICD therapy for VT, and unexpectedly, the non‐inducible patients had a significantly higher cumulative likelihood of ICD treatment for a VF episode [8]. In the PRESERVE EF study, ischemic patients with a LVEF ≧ 40% were enrolled and those with at least one non‐invasive arrhythmic risk factor were referred for EPS. No patients without the risk factors or with risk factors but the non‐inducibility met the primary endpoint of arrhythmic events, thereby yielding the negative predictive value of 100%. The rate of the primary endpoint was about 16% at 2 years in patients with the inducibility [9].

Using the follow‐up data of the nationwide Japan Cardiac Device Treatment Registry (JCDTR) with an implantation date between January 2011 and August 2015, we analyzed consecutive 746 patients who had primary prevention ICD/CRT‐D with a LVEF ≦ 35%. EPS was performed in 118 patients (16% of the population), and the rate of VT/VF induction was 49% [10]. The rate of performing EPS decreased to 6% and the inducibility increased to 62% in the similar patients with an implantation date between January 2018 and September 2020 (presented in 13th Winter Conference on Cardiac Implantable Electronic Devices, the Japanese Heart Rhythm Society, February 5, 2021) The rate of appropriate ICD therapy at 2 years was 19% in the non‐inducible patients and 26% in the inducible patients (*p* = 0.191) [10].

**FIGURE 1:**
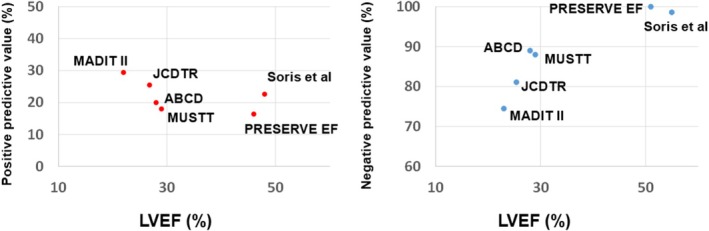
Positive and negative predictive values of VT/VF inducibility by electrophysiological study (EPS) for predicting arrhythmic events, stratified by LVEF. Arrhythmic events were cardiac arrest or death from arrhythmia (MUSTT), [6] appropriate ICD therapy (MADIT II), [8] appropriate ICD therapy or sudden death (Alternans Before Cardioverter Defibrillator [ABCD] trial), [11] sudden cardiac death, VT/VF or appropriate ICD therapy (PRESERVE EF), [9] appropriate ICD therapy (JCDTR), [10] and sudden death, VT/VF or appropriate ICD therapy (Soris et al.) [12]. The values were estimated at 2 years of each study.

Positive and negative predictive values of the VT/VF inducibility by EPS for predicting arrhythmic events at 2 years were plotted against median or mean LVEF in each study population (Figure 1) [6, 7, 8, 9, 10, 11, 12]. Negative predictive value of EPS for predicting arrhythmic events is unsatisfactory in ischemic patients with a LVEF ≦ 35%, whereas it is remarkably high in those with a LVEF ≧ 40%. EPS appears to be a useful tool for arrhythmic risk stratification in ischemic patients with a LVEF ≧ 40%, but not in those with a LVEF ≦ 35%.

## 3 Evidence for Primary Prevention ICD That Deserves Reevaluation

**FIGURE 2:**
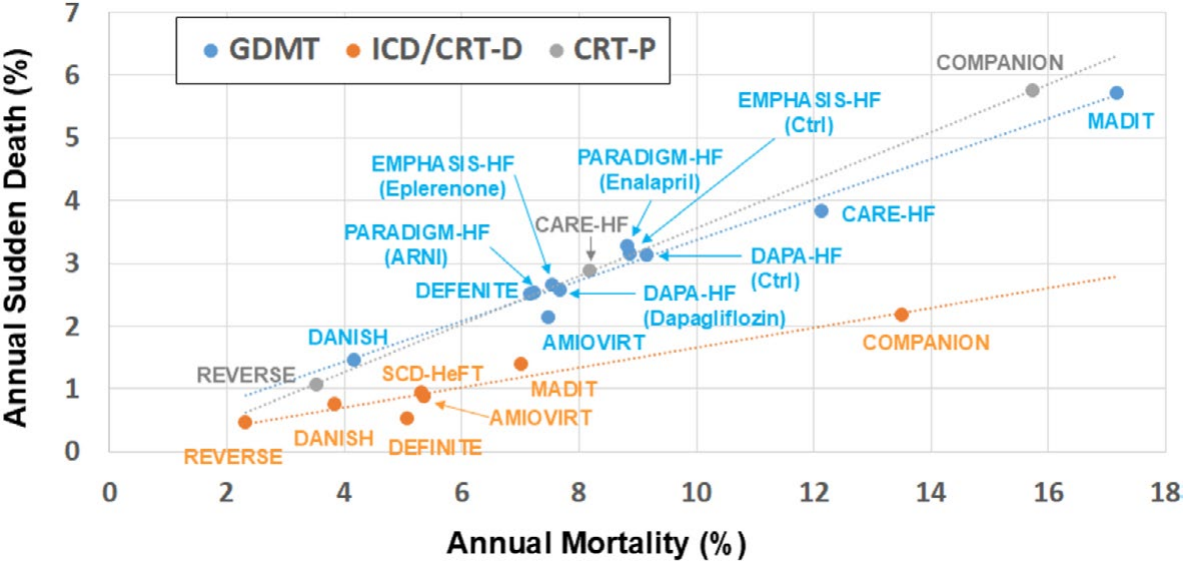
Relationship between the annual rates of sudden death and overall mortality in randomized controlled trials for patients with heart failure with reduced ejection fraction. The slope of guideline‐directed medical therapy (GDMT, blue circle) and that of cardiac resynchronization therapy with a pacemaker (CRT‐P, gray circle) is nearly identical. Defibrillator therapy (ICD/CRT‐D, orange circle) apparently reduces the slope. The slope was 0.32 for GDMT, 0.38 for CRT‐P, and 0.16 for ICD/CRT‐D, respectively. These values indicate that sudden death accounted for 32%, 38%, and 16%, respectively, of the overall mortality in HFrEF patients treated with GDMT, CRT, and ICD/CRT‐D. ARNI: angiotensin receptor neprilysin inhibitor; Ctrl: control; CRT‐D: cardiac resynchronization therapy with a defibrillator; ICD: implantable cardioverter‐defibrillator. Adapted from MADIT [3], AMIOVIRT [18], DEFINITE [19], COMPANION [20], CARE‐HF [21], SCD‐HeFT [22], EMPHASIS‐HF [23], REVERSE [24], PARADIGM‐HF [25], DANISH [5], and DAPA‐HF [26].

Advances in drug therapies such as angiotensin receptor neprilysin inhibitor (ARNI) and sodium‐glucose cotransporter‐2 (SGLT2) inhibitors have reduced a composite of death from cardiovascular causes or hospitalization for heart failure in patients with heart failure with reduced ejection fraction (HFrEF) [13, 14, 15]. In association with the reduced risk of death, rates of sudden death declined substantially over time among ambulatory HFrEF patients who were enrolled in clinical trials, a finding that may argue with a survival benefit from the implantation of an ICD for primary prevention [16]. Sudden death in HFrEF patients was reported in the late 1990s in the Metoprolol CR/XL Randomized Intervention Trial in Congestive Heart Failure (MERIT‐HF) and occurred in 6% per patient‐year in the presence of an angiotensin‐converting enzyme (ACE) inhibitor or angiotensin‐II‐receptor blocker (ARB), which decreased further to 4% in adding metoprolol [17]. Although the rate has been declining with advances in guideline‐directed medical therapy (GDMT), there is a linear relationship between the rates of sudden death and overall mortality, and ICD therapy clearly suppresses this slope (Figure 2) [3, 5, 18, 19, 20, 21, 22, 23, 24, 25, 26].

The efficacy of ICD implantation for primary prevention of sudden cardiac death was conflicting in non‐ischemic HFrEF patients [5, 18, 19, 27]. Especially, the DANISH trial demonstrated that the addition of ICD function fails to prolong survival in those who have LVEF of 35% or less with contemporary GDMT. This would be partly explained by the finding that an optimal age cutoff for ICD implantation for reducing all‐cause mortality was 70 years old or less [28]. With increasing age, the proportion of sudden death diminished progressively [29] and the survival benefit of the ICD was attenuated [30]. Additionally, although the effects of ICD implantation were independent of cardiac resynchronization therapy (CRT) status (*p* = 0.73 for the interaction), the median QRS duration was 146 ms among patients enrolled in the DANISH trial. Therefore, the CRT may have lowered the all‐cause mortality [5]. In fact, for those in NYHA class II with a median QRS duration of less than 130 ms, there was a trend towards a reduction in the long‐term all‐cause mortality rate with ICD implantation (NYHA class II: adjusted hazard ratio [HR] 0.75, 95% confidence interval [CI] 0.56–1.00), when stratified according to CRT status and center [31].

Among patients enrolled in primary prevention ICD trials in the United States, Black patients with non‐ischemic HFrEF were found to be at the highest risk of receiving appropriate ICD therapies (30%, 20%, 25%, and 25% at 3 years for Black non‐ischemic, White non‐ischemic, Black ischemic, and White ischemic patients, respectively) and to be at a similar risk of receiving a first appropriate shock (16%, 11%, 16%, and 16% at 3 years for the same groups, respectively) [32]. It is worth noting that the DANISH trial did not enroll any Black patients [33]. There is heterogeneity in terms of both underlying heart diseases and scar burden among non‐ischemic cardiomyopathies [34, 35, 36]. For example, patient‐related comorbidities were significantly associated with VT/VF recurrences after catheter ablation, being a higher rate in patients with hypertrophic cardiomyopathy, valvular heart disease, and sarcoidosis than those with dilated idiopathic cardiomyopathy [34]. This may be related to the benefits of primary prevention ICD implantation [35, 36].

## 4 Consistent Effect of ICDs on Sudden Death Prevention in Patients With Heart Failure With Reduced Ejection Fraction

Long‐term follow up of SCD‐HeFT patients for up to 11 years still demonstrated that the overall ICD–placebo hazard ratio (HR) for mortality was 0.87 (95% confidence interval [CI]: 0.76 to 0.98; *p* = 0.028) [2]. The contemporary multicenter cohort study (the EU‐CERT‐ICD study), which excluded candidates for CRT device implantation, demonstrated that primary prevention ICD implantation was associated with a 27% lower mortality rate (HR 0.73) in heart failure patients with an LVEF of 35% or less and a narrow QRS [37]. When stratifying by ischemic and non‐ischemic etiology, a higher difference between the ICD group and the control group was observed despite the lower mortality rate in non‐ischemic patients (ischemic: HR 0.79, 95% CI 0.58–1.06, *p* = 0.11; non‐ischemic: HR 0.59, 95% CI 0.38–0.91, *p* = 0.017; *p* = 0.27 for interaction) (Table 1). Similarly, primary prevention ICD/CRT‐D recipients from the Swedish Heart Failure Registry (SwedeHF), with a propensity score‐matched analysis, were associated with reduced short‐ and long‐term all‐cause mortality [38]. ICD implantation of patients with a primary prevention indication was associated with improved mortality, even in the context of evolving adjunctive heart failure treatment. There was no detectable difference in ICD benefit between patients with ischemic and non‐ischemic patients [39]. We also found that primary prevention ICD/CRT‐D implantation reduced the risk of all‐cause mortality in patients with HFrEF eligible for SCD‐HeFT, compared to conventional therapy, in the real‐world cohort [40]. More recently, the primary composite endpoint of all‐cause mortality, advanced heart failure therapy, ventricular arrhythmias, or appropriate ICD shocks was lower in primary prevention ICD/CRT‐D recipients as compared to those without ICD/CRT‐D, with use of SGLT2 inhibitors by 16.0% of the total cohort (17.5% in the ICD/CRT‐D group versus 14.6% in the no ICD/CRT‐D group, *p* = 0.163) [41]. While these cohort studies demonstrated the survival benefit of ICD/CRT‐D in HFrEF patients, [37, 38, 39, 40, 41] the efficacy appeared to be lower for non‐ischemic patients in the latest population [39, 41]. There are also some guideline differences with regard to the recommendation for primary prevention ICD implantation in non‐ischemic HFrEF patients: it is class I in the JCS/JHRS and American guidelines, [42, 43, 44] whereas it is class IIa in the JCS/JHFS and European guidelines [45, 46, 47]. In addition, the survival benefit of ICD/CRT‐D remains uncertain in HFrEF patients who have the contemporary GDMT with optimal achievement of target doses across all heart failure therapies [41].

**TABLE 1:** Cohort studies evaluating the efficacy of primary prevention ICDs/CRT‐Ds in patients with heart failure with reduced ejection fraction.

Study (Published year)	Number of patients	Country or Continent	Follow‐up period	Non‐ischemic etiology	Hazard ratio (95% CI) for all‐cause mortality
Swede HF [38]	ICD/CRT‐D (*n* = 1305) (CRT‐D 34%) No ICD/CRT‐D (*n* = 1305)	Sweden	2.6 years (median)	33%	0.73 (CI 0.60–0.90), *p* < 0.01 ischemic 0.78 (0.63–0.98) Non‐ischemic 0.50 (0.28–0.91) *p*‐interaction 0.17
EU‐CERT‐ICD (2020) [37]	ICD (*n* = 1516) No ICD (*n* = 731)	Europe	2.4 years (mean)	35%	0.73 (0.57–0.94), *p* = 0.014 ischemic 0.79 (0.58–1.06) non‐ischemic 0.59 (0.38–0.91) *p*‐interaction 0.27
Ahmed et al. [39]	ICD (*n* = 8472) No ICD (*n* = 2118)	The United States	2.0 years (mean)	12%	0.76 (0.68–0.85), *p* < 0.001 ischemic 0.76 (0.68–0.85) non‐ischemic 0.62 (0.34–1.15) *p*‐interaction 0.50
JCDTR & JCARE‐CARD (2025) [40]	ICD/CRT‐D (*n* = 285) (CRT‐D 63%) No ICD/CRT‐D (*n* = 285)	Japan	2.0 years (mean)	64%	0.62 (0.40–0.94), *p* = 0.026 ischemic 0.60 (0.32–1.12) non‐ischemic 0.57 (0.33–0.98) *p*‐interaction 0.83
Demir et al. [41]	ICD/CRT‐D (*n* = 588) (CRT‐D 21%) No ICD/CRT‐D (*n* = 683)	Turkey	5.4 years 4.1 years (median)^a^	37%	0.72 (0.13–0.84), *p* < 0.0001^b^ ischemic 0.72 (0.60–0.86) non‐ischemic 0.77 (0.57–1.03) *p*‐interaction not available

Abbreviations: 95% CI, 95% confidence interval; CRT‐D, cardiac resynchronization therapy with a defibrillator; ICD, implantable cardioverter‐defibrillator.

^a^ The median follow‐up period was significantly longer in patients with ICD/CRT‐D (5.4 years) compared to those without ICD/CRT‐D (4.1 years) (*p* < 0.0001).

^b^ In this study, the primary endpoint was a composite of all‐cause mortality, advanced heart failure therapy, ventricular arrhythmias, or appropriate ICD shocks.

## 5 The Effectiveness of Primary Prevention ICD/CRT‐D in Japan

Implantation of ICD/CRT‐D for primary prevention was significantly higher in non‐ischemic patients, including those with dilated and hypertrophic cardiomyopathy, valvular heart disease, and others, than that in ischemic patients; thereby the rate was more than 60% in Japan [48]. More recently, the proportion of non‐ischemic patients among ICD/CRT‐D recipients was similarly over 60% in Japan [49, 50] which is higher than in the United States (US) and other European countries (Table 2). The incidence of appropriate ICD therapy did not differ significantly between primary and secondary prevention in ischemic patients [57, 58, 59] whereas it was higher in secondary than in primary prevention in non‐ischemic patients in Japan [60]. The propensity‐matched analysis from the Nippon Storm Study revealed that the risk of appropriate ICD therapy was significantly higher among non‐ischemic primary prevention ICD/CRT‐D recipients than among ischemic primary prevention ICD/CRT‐D recipients (Table 3) [61].

**TABLE 2:** The proportion of primary prevention ICD/CRT‐D recipients with heart failure with reduced ejection fraction in Japan, the United States, Middle East, Africa, and other European countries.

Study (Published year)	Number of patients	Country or Continent	Proportion of primary prevention	Non‐ischemic etiology
Cohort study				
JCDTR (2020) [50]	*n* = 27 034	Japan	40% (ICD: CRT‐*d* = 65:35)	65%
JCDTR & New JCDTR (2023) [49]	*n* = 22 831	Japan	42% (ICD: CRT‐*d* = 65:35)	66%
CeRtiTude (2015) [51]	*n* = 1170	France	87% (ICD: CRT‐*d* = 0:100)	53%
NIS database (2016) [52]	*n* = 267 901	The United States	72% (ICD: CRT‐*d* = 0:100)	34%
Dhande et al. (2022) [53]	*n* = 6392	The United States	78% (ICD: CRT‐*d* = 65:35)	40%
MEAREE ICD (2025) [54]	*n* = 206	Africa	33% (ICD: CRT‐*d* = 77:23)	50%
	*n* = 227	Middle East	91% (ICD: CRT‐*d* = 54:46)	58%
	*n* = 318	CIS and East Europe	87% (ICD: CRT‐*d* = 81:19)	60%
Randomized controlled trial				
PRAETORIAN (2020) [55]	*n* = 849	The United States and Europe	81% (ICD: CRT‐*d* = 100:0)	31%
PARTITA trial (2022) [56]	*n* = 517	Europe	79% (ICD: CRT‐*d* = 76:24)	22%

Abbreviations: CIS, commonwealth of independent states countries; CRT‐D, cardiac resynchronization therapy with a defibrillator; ICD, implantable cardioverter‐defibrillator; NIS: national inpatient sample.

**TABLE 3:** Incidence rates of first appropriate ICD therapy and ICD shock at 1‐year in primary prevention ICD/CRT‐D recipients with heart failure with reduced ejection fraction in Japan.

Study (Published year) Enrollment year	Number of primary prevention ICD/CRT‐D recipients	Follow‐up period (mean)	Age, years (mean)	LVEF (mean)	Appropriate ICD therapy (at 1‐year)	Appropriate ICD shock (at 1‐year)	All‐cause death (at 1 and 2‐year)
JID‐CAD (2021) [57] 2014–2016	Isch, *n* = 154 (CRT‐D 50%)	2.1 years	70	28%	8%		
JCDTR (2023) [49] 2011–2015	Isch, *n* = 173 non‐isch, *n* = 447 (CRT‐D 100%)	1.8 years	Isch, 70 non‐isch, 66	Isch, 27% non‐isch, 27%	Isch, 6.8% non‐isch, 12% *p* = 0.049		Isch, 13.3%, 23.6% non‐isch, 9.9%, 16.1% *p* = 0.0088
Nippon Storm (2024) [61] 2010–2012	Isch, *n* = 132 non‐isch, *n* = 132 (CRT‐D 70%)	2.2 years	Isch, 69 non‐isch, 68	Isch, 26% non‐isch, 26%	Isch, 9.4% non‐isch, 18% *p* = 0.002	Isch, 1.6% non‐isch, 4.3% *p* = 0.063	Isch, 7.5%, 13.7% non‐isch, 6.7%, 14.7% *p* = 0.103
Hanada et al. [59] 2005–2022	Isch, *n* = 56 (CRT‐D 63%)	5.5 years	68	26%	7.3%		

Abbreviations: ACRT‐D, cardiac resynchronization therapy with a defibrillator; ICD, implantable cardioverter‐defibrillator; Isch, ischemic; LVEF, left ventricular ejection fraction; Non‐isch, non‐ischemic.

**FIGURE 3:**
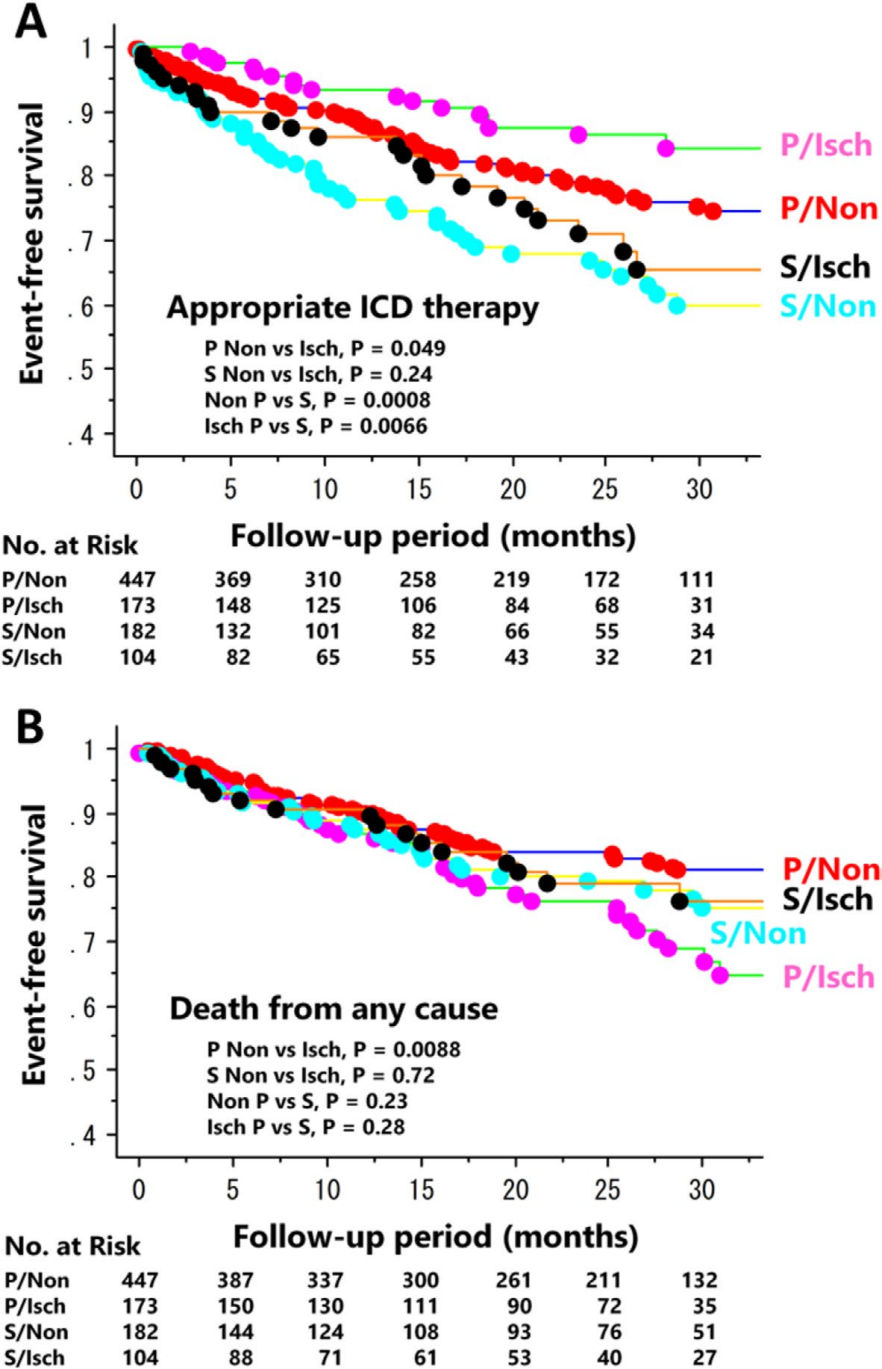
Kaplan–Meier estimates of event‐free survival in CRT‐D recipients stratified by indication and etiology. Outcome events were appropriate ICD therapy (A) and death from any cause (B) P, primary prevention of sudden cardiac death; S, secondary prevention of sudden cardiac death; Isch, ischemic cardiomyopathy; Non, non‐ischemic cardiomyopathy.

Restricting the analysis to CRT‐D recipients using the Japan Cardiac Device Treatment Registry (JCDTR) Database revealed that appropriate ICD therapy occurred more frequently in non‐ischemic patients (19.0%) than in ischemic patients (11.6%) in the primary prevention group, with a mean follow‐up period of 21 months (*p* = 0.049) [49]. However, there was no significant difference in the incidence of appropriate ICD therapy between non‐ischemic (29.1%) and ischemic (23.1%) patients in the secondary prevention group (*p* = 0.24) (Figure 3A). Death from any cause occurred less frequently in non‐ischemic patients (16.1%) than in ischemic patients (24.2%) in the primary prevention group (*p* = 0.0088). There was no significant difference in the incidence of death between non‐ischemic (19.2%) and ischemic (17.3%) patients in the secondary prevention group (*p* = 0.72) (Figure 3B). Therefore, the rate of appropriate defibrillator therapy is likely higher among non‐ischemic than ischemic primary prevention ICD/CRT‐D recipients in Japan. However, we should cautiously interpret the finding because a higher rate of appropriate ICD therapies did not equate to a worse overall prognosis (Table 3) [49, 61] but it was better in non‐ischemic than ischemic patients, as reported previously [1, 2, 62].

## 6 ICD/CRT‐D Utilization in the World and Japan

Risk of sudden cardiac death in HFrEF patients eligible for primary prevention was inversely associated with ICD use in the PARADIGM‐HF trial: 4.5 events/100 person‐years with the lowest ICD use of 1.7% in the Asia‐Pacific region and 1.5 events/100 person‐years with the highest ICD use of 54% in the North America region [63]. The high use of primary prevention ICD in North America can be attributable to the nationally covered ICD indications for patients eligible for the MADIT‐II and SCD‐HeFT in Medicare and Medicaid Services (https://www.cms.gov/medicare‐coverage‐database/view/ncd.aspx?ncdid%20=%20110&ncdver%20=%205, accessed November 25, 2025). The Task Force members of the European Society of Cardiology developed a set of quality indicators. One indicator is the proportion of patients with ischemic cardiomyopathy, NYHA class II‐III who have EF ≤ 35% despite ≥ 3 months of optimal medical therapy and life expectancy > 1 year who receive ICD for primary prevention of sudden cardiac death [64].

**FIGURE 4:**
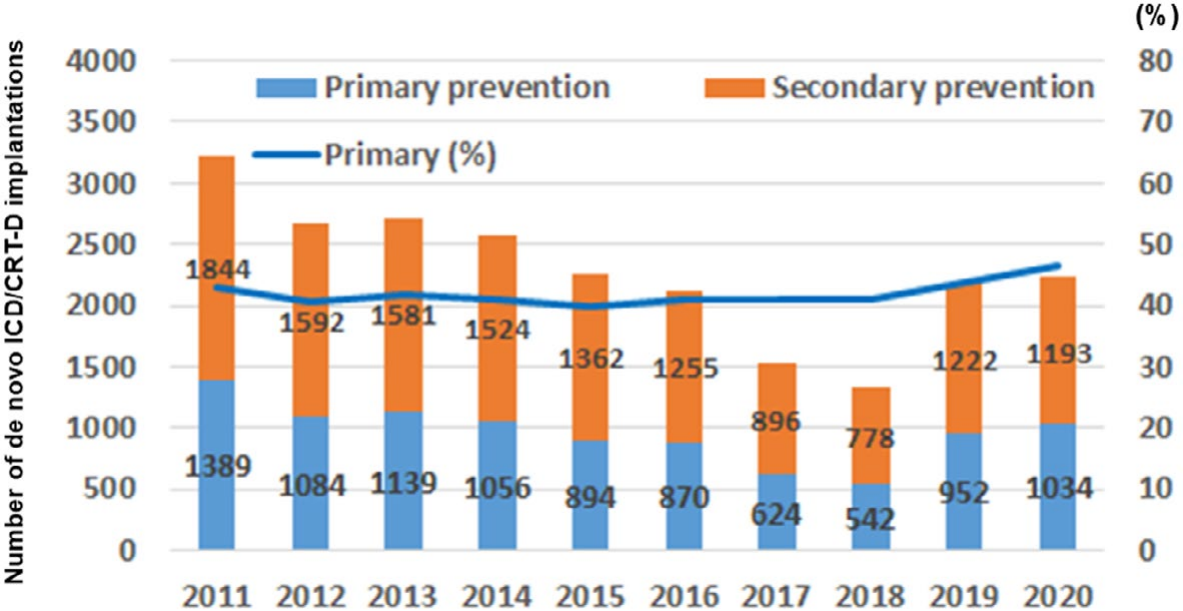
Annual trends in the number of patients with primary and secondary prevention ICD/CRT‐D implantations with the proportion of primary prevention registered in the JCDTR and New JCDTR. Absolute number of patients with primary prevention ICD/CRT‐D implantations and those with secondary prevention ICD/CRT‐D implantations is given by blue bars and orange bars, respectively. The proportion of primary prevention ICD/CRT‐D implantations (a dark blue line) is 40% range between 2011 and 2020. Adapted from [49].

In Japan, it was reported that only about 30% of HFrEF patients meeting the Class I indication for primary prevention ICD/CRT‐D therapy according to the JCS/JHRS guideline recommendations actually received ICD/CRT‐D treatment [65]. Furthermore, of those meeting Class IIa indications, only around 7% underwent ICD/CRT‐D implantation. Consequently, about 40% of ICD/CRT‐D recipients were for primary prevention (Figure 4) [49]. This figure is far lower than in the United States, Middle East, and other European countries, but similar to that in Africa (Table 2).

**FIGURE 5:**
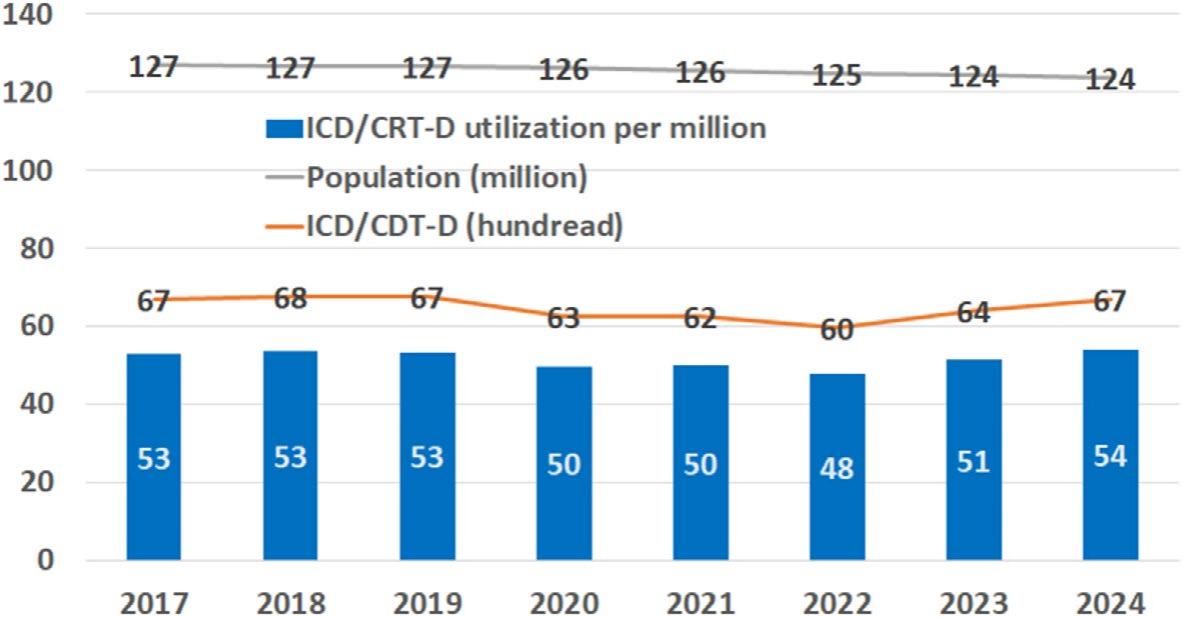
Annual trends of ICD/CRT‐D utilization in Japan. A gray line indicates Japanese population (million) (https://www.stat.go.jp/data/jinsui/2023np/index.html, accessed November 25, 2025). An orange line indicates the number of de novo ICD/CRT‐D implantation (hundred) (Japan Arrhythmia Device Industry Association, https://www.jadia.or.jp/medical/crt‐d.html, accessed November 25, 2025). The ICD/CRT‐D utilization per million in Japan is given by blue bars.

**FIGURE 6:**
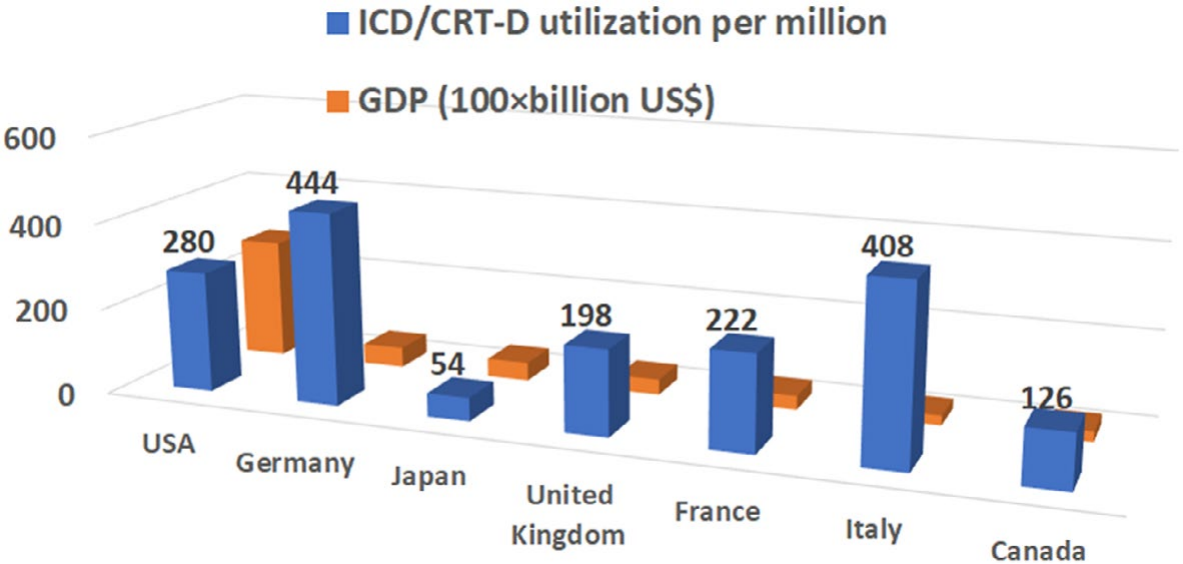
ICD/CRT‐D utilization and Gross Domestic Product (GDP) in the Group of Seven (G7) countries. ICD/CRT‐D utilization (dark blue bars) was expressed as the number of de novo ICD/CRT‐D implantation per million in each country. Adapted from Patel MJ et al. [67], Raatikainen MJP et al. [68], and Canada's Drug Agency, Implantable Cardioverter‐Defibrillator Decision‐Making, (https://www.cda‐amc.ca/implantable‐cardioverter‐defibrillator‐decision‐making, accessed November 25, 2025). Orange bars indicate Gross Domestic Product (GDP) in 2024 (https://eleminist.com/article/3258, accessed November 25, 2025).

In 2018, the number of de novo ICD/CRT‐D implantation reached a maximum of 6772 in Japan. With a population of 124 million, Japan's ICD/CRT‐D utilization rate was calculated to be 54 per million in 2024. The utilization of ICDs/CRT‐Ds in Japan has remained unchanged for several years (Figure 5). Among the Group of Seven (G7) countries, Japan had the third highest Gross Domestic Product (GDP), whereas it had the lowest ICD/CRT‐D utilization. Japan's ICD/CRT‐D utilization rate of 54 per million is the same as the lowest level in European countries in 2005 [66] (Figure 6).

## 7 Current Status of Out‐Of‐Hospital Cardiac Arrest in Japan and Its Impact on Healthcare Costs: Does ICD Therapy Curb Healthcare Expenditures?

**FIGURE 7:**
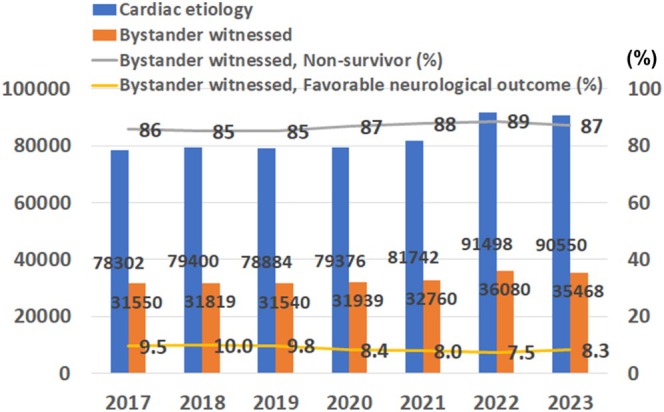
Annual trends of out‐of‐hospital cardiac arrest (OHCA) with cardiac etiology and the outcomes in Japan. Dark blue bars show the absolute number of victims of cardiac OHCA, while orange bars show the absolute number of victims with bystander‐witnessed cardiac OHCA. A gray line indicates the proportion of non‐survivors among victims of bystander‐witnessed cardiac OHCA. A yellow line shows the proportion of victims with favorable neurological outcomes. Adapted from https://www.fdma.go.jp/publication/#rescue, accessed November 25, 2025.

There has been an increasing trend in sudden cardiac death in Japan, with over 90 000 cases recorded in 2023 (Figure 7) (https://www.fdma.go.jp/publication/#rescue, accessed November 25, 2025). As approximately 80% of cases of sudden cardiac death are reported to be due VT or VF, [69, 70, 71] and over 40% of victims of out‐of‐hospital cardiac arrest (OHCA) with cardiac etiology were eligible for primary prevention ICD implantation, [72] nearly 30 000 cases might have been potential candidates for prophylactic ICD therapy in accordance with the JCS/JHRS guidelines [43]. This number is not so surprising, as was estimated that the annual number of sudden death in heart failure patients eligible for SCD‐HeFT represented about 100 000 in the United States [73]. If we had implanted additional 30 000 ICD/CRT‐D implantations in 2024, the ICD/CRT‐D utilization rate would have been 296 per million in Japan. This figure would be similar to that in the United States, but still lower than that in Germany and Italy (Figure 6).

Even when restricted to cases witnessed by bystanders, the rate of return to social activity with favorable outcomes (Cerebral Performance Category Score 1–2) [74] was 8.3% (Figure 7) (https://www.fdma.go.jp/publication/#rescue, accessed November 25, 2025). Accordingly, we unfortunately lost more than 82 500 people to sudden cardiac death or unfavorable neurological outcomes in 2023, which would require a significant amount of medical resources and result in a labor shortage.

In the Japanese healthcare context, the incremental cost‐effectiveness ratio (ICER) was US $29 838 (¥4 364 106) per quality‐adjusted life year (QALY), which was below the reference value of ¥5 000 000. However, depending on several assumptions, the ICER may exceed the reference value. Sensitivity analyses highlighted the significant impact of the hazard ratio and battery longevity on cost‐effectiveness [75]. Based on the results from our cohort study, [40] the number needed to treat (NNT) was calculated to be 16 and 15 for prevention of one all‐cause death and one sudden cardiac death at 3 years. The NTT for prevention of all‐cause death was 18 in the MADIT‐II and 13 in the SCD‐HeFT [76].

According to the Diagnosis Procedure Combination database of the Japanese Registry of All Cardiac and Vascular Diseases (JROAD‐DPC), the number of victims of cardiogenic OHCA increased from 22 000 in 2012 to nearly 40 000 in 2022. Naturally, the cost of hospitalization increased from ¥13 billion in 2012 to ¥24 billion in 2022, most of which was spent on patients who ultimately died or experienced unfavorable neurological outcomes. The ¥24 billion in 2022 corresponded to the cost of de novo ICD implantation in 4800 patients (the cost of planned ICD implantation was approximately ¥5 000 000), which could have covered about 75% of the annual cost of ICD/CRT‐D implantations in Japan (Watanabe M, in preparation).

## 8 Conclusions

The DANISH trial raised questions about the effectiveness of primary prevention ICD/CRT‐D in patients with non‐ischemic HFrEF. Conversely, several studies have shown a survival benefit from ICD/CRT‐D in non‐ischemic HFrEF patients. The heterogeneity of non‐ischemic cardiomyopathy may explain the discrepancy in findings between the DANISH trial and other studies.

In Japan, non‐ischemic etiology is prevalent among candidates for ICDs/CRT‐Ds, and ICD/CRT‐D utilization is the lowest among G7 countries. It is time to revise the Japanese restriction of health insurance coverage for primary prevention ICD/CRT‐D recipients, for which induction of VT/VF with an electrophysiological study is generally required. Additionally, cardiac electrophysiologists should participate in educational activities that emphasize the importance and indications for primary prevention ICD/CRT‐D implantation. Given Japan's limited medical resources, we must also identify HFrEF patients at higher risk for sudden cardiac death with low risk for non‐cardiac death to ensure the cost‐effectiveness of primary prevention ICD/CRT‐D implantation. In this regard, we anticipate the results of the PROFID EHRA randomized clinical trial [77].

## Funding

The authors have nothing to report.

## Ethics Statement

The study was approved in the Ethics Committee of Sapporo City General Hospital on May 16, 2018 (Approval No.:H30‐057‐455).

## Consent

Patient consent has been obtained in an opt‐out manner in Sapporo City General Hospital. There is no clinical trial registration number regarding the study.

## Conflicts of Interest

The authors declare no conflicts of interest.

## Data Availability

The data that support the findings of this study are available on request from the corresponding author. The data are not publicly available due to privacy or ethical restrictions.
